# *Trypanosoma cruzi*: Role of δ-Amastin on Extracellular Amastigote Cell Invasion and Differentiation

**DOI:** 10.1371/journal.pone.0051804

**Published:** 2012-12-18

**Authors:** Mário C. Cruz, Normanda Souza-Melo, Claudio Vieira da Silva, Wanderson Duarte DaRocha, Diana Bahia, Patrícia R. Araújo, Santuza R. Teixeira, Renato A. Mortara

**Affiliations:** 1 Departamento de Microbiologia, Imunologia e Parasitologia, Escola Paulista de Medicina–UNIFESP, São Paulo, São Paulo, Brazil; 2 Departamento de Bioquímica e Biologia Molecular, Universidade Federal do Paraná, Curitiba, Paraná, Brazil; 3 Disciplina de Imunologia - Instituto de Ciências Biomédicas, Universidade Federal de Uberlândia, Campus Umuarama, Uberlândia, Minas Gerais, Brazil; 4 Departamento de Bioquímica e Imunologia, Universidade Federal de Minas Gerais, Pampulha, Belo Horizonte, Minas Gerais, Brazil; University of Oklahoma Health Sciences Center, United States of America

## Abstract

*Trypanosoma cruzi* is a protozoan parasite that comprises different phylogenetic groups and is the causative agent of Chagas’ disease. Different *T. cruzi* strains present differences in infectivity in *in vitro* and *in vivo* experimental models, which are likely related to the expression of different virulence factors. Amastin is a surface glycoprotein abundantly expressed on the intracellular mammalian amastigote form of the parasite. In this study, we showed that a highly infective strain (G strain) of extracellular amastigote (EA) invasive forms expressed reduced RNA levels of amastin compared to a less infective strain (CL). The treatment of HeLa cells with recombinant δ-amastin reduced infectivity of EA forms. However, the ectopic expression of δ-amastin accelerated amastigote differentiation into trypomastigotes. Corroborating the virulence behavior in association with amastin expression, the EAs overexpressing amastin were precociously and robustly observed in the liver of susceptible mouse strains (A/JUnib), whereas parasitemia was never detected in *in vivo* assays. This is the first report on the regulatory role of amastin in the course of both *in vitro* and *in vivo T. cruzi* infection.

## Introduction

*Trypanosoma cruzi*, the flagellated protozoan that causes Chagas disease in the Americas, is able to invade different mammalian cells in order to complete its life cycle [1], [2]. Metacyclic and bloodstream trypomastigote forms are the classic infective forms that initiate and spread infection between hosts [3]. Trypomastigotes of distinct *T. cruzi* isolates have been shown to possess highly distinct levels of infective capacity towards cultured mammalian cells and in animal models [4]–[6]. Amastigotes generated (extracellular amastigotes or EAs) by the extracellular differentiation of bloodstream or the corresponding tissue culture derived trypomastigotes are also capable of sustaining an infective cycle in the mammalian host and cells [7]–[10].

EA forms of some *T. cruzi* strains (such as G and CL strains) display the opposite pattern of infectivity of trypomastigote forms [11], [12]. Whereas both metacyclic and tissue culture derived trypomastigotes from G strains exhibit very low infectivity both *in vitro* and *in vivo* [6], EAs are highly infective *in vitro* [11], [12]. By contrast, little is known regarding the elements involved in EA entry into mammalian cells [reviewed in 13]. Carbohydrate epitopes expressed on the surface of EA have been shown to play a role in entry, probably in the initial steps of parasite attachment [12]. A 21 kDa protein expressed in all developmental stages of the parasite up-regulates cell invasion by EAs and metacyclic trypomastigotes [14]. However, the molecular basis of the remarkable capacity of EA parasites to invade mammalian cell *in vitro* still remains unknown.

Other groups have studied amastigote specific factors involved in intracellular infection. The involvement of molecules such as Asp-1 and Asp-2 in colonization of host cells as well as protective immunity has been experimentally demonstrated [15]–[17]. Also, mannose residues on transialidase-like molecules in amastigotes have been implicated in their invasion of macrophages through mannose receptors [18].

It is conceivable that the *T. cruzi* protein repertoire changes in a stage-specific manner, with up- and down-regulation of several factors involved in the exacerbation or arrest of intracellular infection. This repertoire varies from strain to strain, as proteins are isolated from different host organisms, and serves as different evasive/infective parasite factors [19].

One of the families of *T. cruzi* surface proteins is the amastin multi-gene family, which consists of small proteins of about 200 amino acids and was first identified by its higher expression in amastigotes from the Tulahuen strain [20] There are also approximately 45 members of the amastin gene family dispersed throughout the genome of all *Leishmania* species, showing different expression patterns [21], [22]. By contrast, the amastin gene family is reduced in the *T. cruzi* genome, but is present in all tested *T. cruzi* strains [23]. Some of its members are organized in large clusters containing alternating copies of tuzin genes. Phylogenetic analysis of trypanosomatid amastins defined four subfamilies (α, β, γ and δ) with distinct genomic organization as well as patterns of expression during the cell cycle of *T. cruzi* and *Leishmania* spp. [24].

The amastin N-terminal signature peptides are among the most immunogenic of all leishmanial surface antigens in mice [25] and generate strong immune responses in humans with visceral leishmaniasis [26]. Thus, amastin proteins seem to operate at the host–parasite interface and are likely to be involved in disease prognosis. The putative role of amastins in intracellular survival has been suggested by DNA microarray analysis data, which indicates that amastin genes are predominantly expressed in *L. donovani* amastigotes of different isolated from patients with post-kala-azar dermal leishmaniasis and visceral leishmaniasis [21], and was also shown for laboratory strains [28]. Although the exact role of amastin proteins in disease progression has not yet been determined and the biological function of amastins remains unknown, it has been hypothesized that *Leishmania* amastins may play a role in proton or ion traffic across the cell membrane to adjust cytoplasmic pH under the harsh conditions of the phagolysosome [21].

In this study, we report for the first time both *in vitro* and *in vivo* roles for amastin in *T. cruzi*-host interplay. By using both recombinant δ-amastin protein and transgenic parasites overexpressing amastin in *in vitro* and *in* *vivo* models, we were able to demonstrate that recombinant amastin can adhere to host cells and inhibit mammalian cell invasion by *T. cruzi* EAs. Constitutive overexpression of a δ-amastin in the G strain led to enhanced differentiation into metacyclic trypomastigotes. Interestingly, although parasites (EAs) overexpressing δ-amastin had a lower infection capacity *in vitro*, they differentiate faster *in vitro* into trypomastigotes and, correspondingly, their amastigote nests are detected very early during *in vivo* infections.

## Materials and Methods

### Parasites, Mammalian Cells and Invasion Assays

G and CL *T. cruzi* strains were used in the study according to [12]. The CL Brener *T. cruzi* strain [29] was also used in this study. EAs were obtained after differentiation of tissue culture trypomastigotes (TCTs) in LIT medium as previously described [12]. Epimastigotes were obtained as previously described [14].

Vero and HeLa cells (obtained from Instituto Adolfo Lutz, São Paulo, SP, Brazil) were cultured in Dulbecco’s minimal essential medium (DMEM) (Sigma Chemical Co., St. Louis, MO, USA) supplemented with 10% fetal bovine serum (FBS, Cultilab, Campinas, SP, Brazil), 10 µg/mL streptomycin (Sigma, USA), 100 U/ml penicillin (Sigma, USA) and 40 µg/mL gentamycin (Sigma, USA) at 37°C in a 5% CO_2_ humidified atmosphere.

HeLa cell invasion assays were performed in 24 wells plates containing sterile glass coverslips in which 500 µL of cell suspension (2×10^5^ cells) were added to each well to seed overnight. EA suspensions (10 parasites/cell) were added and the plates were incubated for 2 h at 37°C in a CO_2_ (5%) humidified incubator. After incubation, the cells were gently washed eight times with PBS, fixed with Bouin’s reagent and stained with Giemsa stain [30].

Cell invasion assays in the presence of recombinant amastin were performed by treating host cells with 5 µg/mL of GST (glutatione S-transferase)-δ-AmastinH or GST for 1 h before addition of parasites.

### Cloning and Purification of Recombinant GST-AmastinH

A region of a delta-amastin gene (GenBank XP_812391) encoding for a hydrophilic portion of the protein (AmastinH) was chosen to be cloned and expressed in fusion with a GST tag in the plasmid pGEX-4T2. A DNA fragment derived from the CL Brener strain total genomic DNA was amplified by PCR using the following primers: forward BamHI-5′AAG GAT CCC TGG TTG GGA CGC CGA TAG ACC AG 3′ and reverse BamHI -5′AAG GAT CCA CAT TCA CGA AAA TCT TCC CAA AA 3′. The insert was cloned into the plasmid pGEX-4T2 (GE Healthcare, USA) and the plasmid was used to transform *Escherichia coli* BL-21 to produce recombinant GST-δ-AmastinH. Recombinant GST-δ-AmastinH was purified using glutathione (GSH) agarose beads (Pierce, USA) then dialyzed against PBS for 48 h at 4°C. The amount of purified protein was determined using the Coomassie Plus assay reagent (Pierce, USA) and measuring the optical density at 620 nm, and the purified protein was analyzed by Coomassie Blue stained SDS-polyacrylamide gels.

### Real-time Quantitative PCR

RNA was extracted from parasites with TRIzol® Reagent and treated with RNase-free DNase (Invitrogen, USA). First strand cDNA was synthesized using the ThermoScript™ Real-Time PCR System according to the manufacturer’s instructions (Invitrogen, USA). Specific forward and reverse primers designed based upon the nucleotide sequences of the amastin gene were used to amplify sequences of this gene by PCR. Quantitative real-time PCR (qRT-PCR) was performed with 2.0 µL of the cDNA reaction in 20 µL of SYBR® Green PCR Master Mix (Applied Biosystems, Foster City, CA, USA) with the primers described below (500 nM). Amastin cDNA was measured using the oligonucleotides Amastin-Forward 5′ GGC GGC ACA CTT CTA CCT AA 3′ and Amastin-Reverse 5′ ACA ATG CTG ACC ACC AAC AG 3′. GAPDH cDNAs (used as an internal control) were measured with the following oligonucleotides: GAPDH-Foward 5′ AGC GCG CGT CTA AGA CTT ACA 3′ and GAPDH-Reverse 5′ TGG AGC TGC GGT TGT CAA TT 3′. The reactions were carried out with the ABI Prism® SDS 7000 (Applied Biosystems, Foster City, CA, USA) and analyzed with the associated software (version 2.0) using the standard protocol. The primers were designed to achieve maximum polymerase efficiency. Each amplicon was about 75 bp in length and each reaction was repeated three to five times to calculate the standard deviations. The comparative mRNA levels were determined after normalization to glyceraldehyde-3-phosphate dehydrogenase (GAPDH) amplicons.

### Northern and Western Blot Assays

Total RNAs isolated from epimastigote, trypomastigote and amastigote cultures were separated using the RNeasy® kit (Qiagen, USA), transferred to Hybond-N+ membranes and hybridized with the amastin open reading frame (U04339) as described [31]. For the western blot, wild-type and stably transfected epimastigote cell lysates were prepared by homogenization of cell pellets in Laemmli sample buffer without boiling. Proteins were separated in 12.5% standard SDS-polyacrylamide gels, transferred to Hybond-C membranes (Amersham Pharmacia Biotech, USA), and blocked with 5% milk-PBS-Tween 0.1%. Membranes were then incubated with mouse anti-GFP (Santa Cruz Biotechnology, USA), washed, and then incubated with secondary antibody (goat anti-mouse (H+L)-HRP conjugate, Bio-Rad, USA) as previously described [32]. The immunocomplexes were detected using the enhanced chemiluminescent substrate, ECL-Plus (Amersham Pharmacia Biotech, USA) according to instructions provided by the manufacturer.

### Flow Cytometry

Approximately 1×10^7^ live parasites were washed with cold PBS and analyzed using a BD FACSCalibur™ flow cytometer (Becton Dickinson, USA) with 10^4^ gated events acquired for analysis. Untransfected control parasites, which showed intrinsic fluorescence, were used to establish the cut-off value.

### Plasmid Constructions and Parasite Transfections

To overexpress δ-amastin in *T. cruzi* G strain, the coding sequence of the TcA21 cDNA clone [20; accession number U04339] was PCR-amplified using a forward primer (5′-CATCTAGAAAGCAATGAGCAAAC-3′) and a reverse primer (5′-CTGGATCCCTAGCATACGCAGAAGCAC-3′), which contained *Xba*I and *Bam*HI restriction sites, respectively. It was then digested with *Xba*I and *Bam*HI (the sites underlined in the primers above), treated with shrimp alkaline phosphatase (SAP) and ligated into a *Bam*HI/*Xho*I GFP fragment derived from pTREX-GFP [33]. The ligation product was purified and digested with *Xba*I/*Xho*I, followed by purification and cloning in the pTREX vector [33] at the same sites, generating the pTREX-δ-Amastin-GFP. The plasmids created were checked by DNA sequencing.

Epimastigotes were harvested from cultures, washed once with PBS and resuspended to 2×10^8^ parasites/ml in electroporation buffer (137 mM NaCl, 21 mM HEPES, 5 mM KCl, 5.5 mM Na_2_HPO_4_, 0.77 mM glucose, pH 7.0). Aliquots (0.7 ml) of parasite suspension were mixed with 25 µg DNA in 0.4 cm cuvettes and electroporated using a Bio-Rad Gene Pulser® set at 0.3 kV and 500 µF with two pulses. The transfected cells were transferred to 5 ml of LIT with 10% FCS and incubated at 28°C for 48 h before adding G418 (500 µg/ml). Plasmid DNA used in electroporation experiments was obtained by alkaline lysis using Qiagen columns (Qiagen, USA).

### GST-δ-AmastinH: Antibodies and Cell Binding Assay

Antibodies were produced in eight week old rabbits immunized with GST-δ-AmastinH. Rabbits received the first dose of antigen (400 µg) adsorbed in complete Freund’s adjuvant (Pierce, USA) and after two weeks received three additional doses of the antigen plus incomplete Freud’s adjuvant at two week intervals. Ten days after the last immunizing dose, rabbits were bled by heart puncture, and serum was collected and stored at −20°C until usage. HeLa cells (5 × 10^4^) were placed in 96-well microtiter plates and were grown overnight at 37°C. Live cells were washed in PBS and blocked with PBS containing 10% FCS (PBS-FCS) for 1 h at room temperature. Increasing amounts of purified recombinant GST-δ-AmastinH or GST were added to the wells, and the incubation proceeded for 1 h at 37°C. Cells were then fixed with 4% paraformaldehyde in PBS. To detect bound amastin on the surface of HeLa cells, immunostaining of δ-AmastinH was performed as described [14] using the rabbit antibodies described above.

### Immunofluorescence

Coverslips containing infected cells were washed with PBS, fixed with 3.5% formaldehyde in PBS for 1 h, washed three times with PBS and then permeabilized with 0.1% saponin (BDH, Amersham, UK) in PBS containing 0.2% gelatin and 0.1% NaN_3_ (PGN). The coverslips were incubated with Anti-GST-δ-AmastinH (diluted 1∶50 in PGN) for 1 h at room temperature, washed three times with PBS and then incubated with fluorescein-labeled goat anti-rabbit IgG (Sigma, USA) diluted 1∶100 in PGN for 1 h in the presence of 10 µM 4′,6-diamidino-2-phenylindole dihydrochloride (DAPI, Molecular Probes, Eugene, OR, USA). After three washes with PBS, the coverslips were mounted in glycerol buffered with 0.1 M Tris, pH 8.6 and 0.1% paraphenylenediamine to reduce photobleaching. Images were acquired with an Olympus BX51 under an epifluorescence microscope. Confocal images were obtained using a Bio-Rad 1024UV system coupled to a Zeiss Axiovert 100 microscope or a Leica TCS SP5 II system. Images were acquired with 100× (1.4 NA) oil immersion objectives.

### Intracellular Growth and Metacyclogenesis

HeLa cells were infected with wild type G strain, G-pTREX-GFP and G-pTREX-δ-Amastin-GFP for 24, 48 and 72 h, fixed with Bouin and stained with Giemsa as described above for intracellular growth counting in triplicate coverslips.

Epimastigotes that had been cultured for 6 days (5×10^7^ cells/ml) were incubated at 28°C in LIT medium with 5% FCS for various time periods. The relative numbers of metacyclic trypomastigotes were morphologically determined by counting in a Neubauer chamber over 23 days.

### Animals and Histological Analysis

Six week old A/JUnib mice were used for *in vivo* infection, as this strain has been previously shown to be highly susceptible to *T. cruzi* infection [34]. All experiments involving animal work were conducted under guidelines approved by the UNIFESP ethics committee, which are in accordance with international recommendations. We inoculated 5×10^6^ extracellular amastigotes of G-pTREX-GFP and G-pTREX-δ-Amastin-GFP strains intravenously in a volume of 200 µL. Tissue parasitism was quantified from day 2 p.i. by examining the liver of mice belonging to each of the two groups (15 animals/group). Liver, spleen, heart and kidney from all animals were fixed in 10% formaldehyde in 0.1 M phosphate buffer (pH 7.3) for 24 h, then dehydrated in ethanol, clarified in xylene, embedded in paraffin, and 5 µm thick sections were obtained from each block. Paraffin sections were stained with hematoxylin–eosin for routine histological analysis. Amastigote nests were quantified in 100 microscopic fields/section in triplicate slides, as previously described [47].

### Statistics

All experiments were performed with duplicate coverslips and repeated at least three times. Three hundred cells per coverslip were analyzed. Statistical analysis was performed by SigmaStat (Version 1.0, Jandel Scientific), employing Student’s *t*-test. Data are presented as mean +/− standard deviation (SD).

### Ethics Statement

All experiments involving animal work were conducted under Brazilian National Commitee on Ethics in Research (CONEP) ethic guidelines, which are in accordance with international standards (CIOMS/OMS, 1985). The present study was approved by CEP/UNIFESP (Comitê de Ética em Pesquisa da Universidade Federal de São Paulo/Hospital São Paulo) under the protocol number 1839/07.

## Results

### Distinct *T. cruzi* strains (CL and G) Express Different Levels of Amastin Transcripts

**Figure 1:**
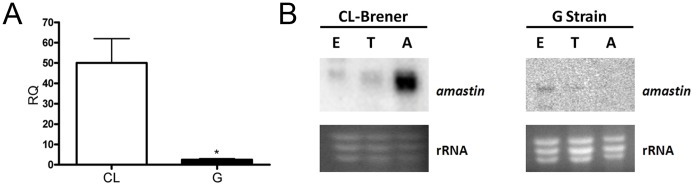
Delta-amastin is more abundant in less infective *T. cruzi* extracellular amastigotes. A. **mRNAs in EAs of the G strain are less abundant when compared to EAs of the CL-Brener clone** ***T. cruzi*** **.** Transcript levels were determined by quantitative real-time PCR using SYBR® Green I chemistry. qRT-PCR was performed on RNA samples from EAs of G and CL strains. The comparative mRNA levels were determined after normalization with GAPDH amplicons. Standard deviations are derived from three replicates. *p<0.05 **B. mRNA corresponding to amastin is preferentially expressed in amastigotes from CL Brener clone.** Northern blot analyses of total RNA (10 µg) from *T. cruzi* epimastigotes (E), trypomastigotes (T) and amastigotes (A) from CL-Brener clone or the G *T. cruzi* strain was submitted to electrophoresis and blotted on nylon membranes by standard procedures. Each blot was hybridized with amastin probe previously labeled with [α-^32P^]-dCTP. To determine equal loading of RNA, the 1.2% agarose/MOPS/formaldehyde gel was stained with ethidium bromide (bottom panel).

To evaluate amastin expression in EAs from CL and G strains, the quantity of transcript in each strain was determined by quantitative real-time PCR using primers designed to achieve the maximum polymerase efficiency and GAPDH amplicons as a reference. The level of amastin transcripts was ∼60 fold higher in EAs from the CL strain (less infective) as compared to EAs from the G strain (more infective) (Fig. 1A). However, this expression data could be underestimated in the G strain since the primers used were designed based on the CL Brener clone genomic sequence. To circumvent this bias, total RNA from each strain was probed by northern blot assays using an amastin coding sequence (Fig. 1B). This approach confirmed the quantitative real-time PCR data.

### Recombinant δ-amastin Adhered to Host Cells

**Figure 2:**
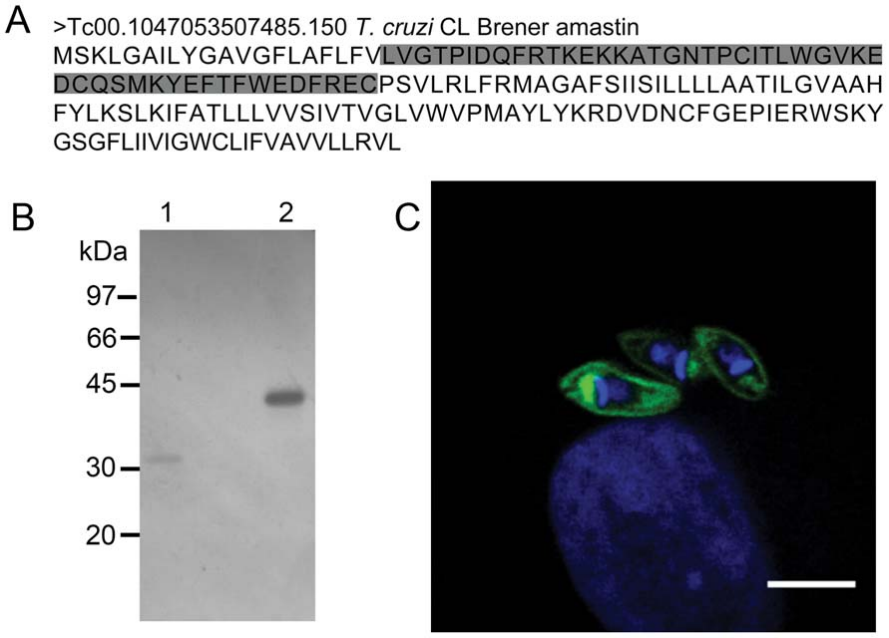
Reactivity of anti-recombinant δ-amastin antibodies. **A: Representation of full length amastin amino acids:** The sequence illustrates the expressed δ-amastinH region (gray/boxed) cloned in fusion with GST to produce the recombinant protein GST-δ-AmastinH. **B:** **Western blot analysis with polyclonal antibodies recognized GST-δ-AmastinH protein. 1:** Purified GST (5 µg) or **2:** GST-δ-AmastinH (5 µg) revealed with anti-GST-δ-AmastinH confirmed the efficient reactivity of the polyclonal antibody with the recombinant protein and GST. **C.** **Surface localization of amastin defined by polyclonal rabbit anti-GST-AmastinH.** Immunofluorescence of intracellular amastigotes of G strain with rabbit anti-GST-δ-AmastinH (green) and DAPI (blue). Image obtained by confocal microscopy. (Bar = 3 µm).

A previous report described that several attempts to express full length amastin failed, probably due to its toxicity to *E. coli* [20]. Here, we chose a region encoding the first hydrophilic portion of the protein (shown in Fig. 2A, in gray to clone and express in fusion with GST -GST-δ-AmastinH). The antibody raised in rabbit against this hydrophilic region of amastin specifically recognizes the recombinant amastin as well as GST, although, as shown in Fig. 2B, the reactivity is stronger with GST-δ-AmastinH. To confirm its specificity, we carried out immunostaining of G strain amastigotes inside of Vero cells four days after of infection with TCTs and observed a significant labeling of the parasite membranes as well as intracellular components in the vicinity of the kinetoplast (Fig. 2C). The specificity of the antibody was further confirmed by immuofluorescence with epimastigotes and metacyclic trypomastigotes (figure S1). The result highlights that the anti-GST-δ-AmastinH recognizes the amastin at the cell surface of amastigotes, but not in epimastigotes or trypomastigotes (figure S1). Further controls were carried out and confirmed the specificity of the antibodies used in this study (figure S2).

**Figure 3:**
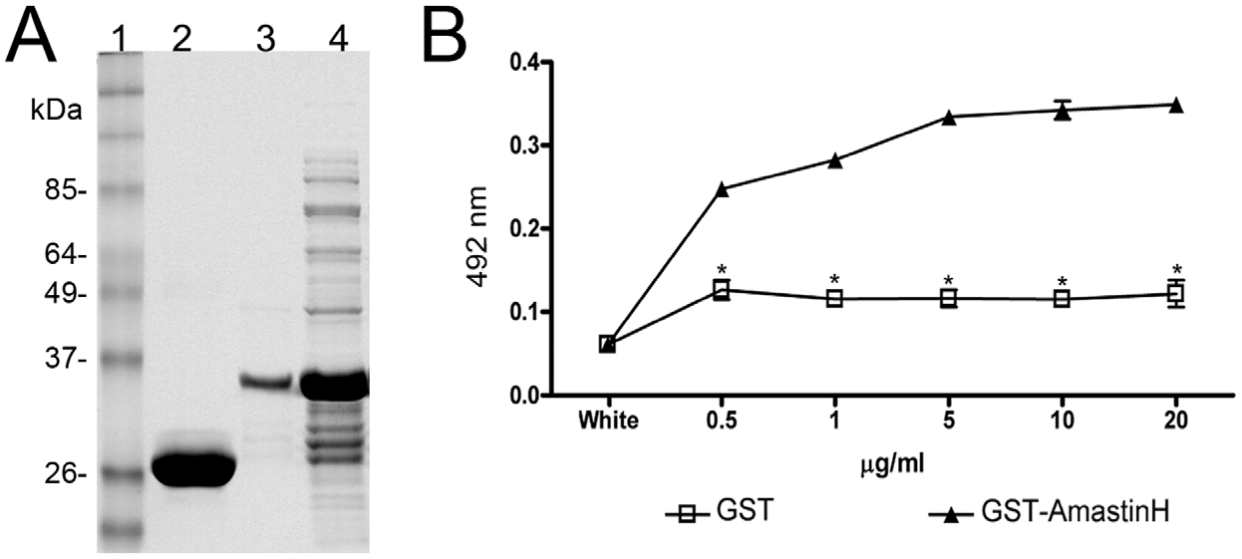
GST-δ-AmastinH specifically binds to HeLa cells in a dose-dependent saturable manner. **A. SDS-PAGE showing the purity of recombinant GST-δ-amastinH:** 1; Molecular weigh markes, in kiloDaltons; 2: GST alone; 3: purified recombinant GST-δ-AmastinH; 4 total extract of induced *E. coli*. **B: Recombinant GST-δ-AmastinH binds to HeLa cells**. Increasing concentrations of GST-AmastinH or GST (negative control) were added to wells in ELISA plates containing adhered and fixed HeLa cells. After washing, cells were sequentially incubated with anti-GST antibodies and anti-rabbit IgG conjugated to peroxidase. The bound enzyme was revealed by *o-*phenylenediamine as a substrate. Representative results of two independent experiments are shown. *p<0.05.

Based on amastin topology prediction by Rochette et al. in 2005 [21], the hydrophilic region chosen in this study would be exposed to the extracellular environment. This arrangement prompted us to test if this polypeptide could interact with the host cell. Therefore HeLa cells grown on microtiter plates were treated with increasing concentrations of GST-δ-AmastinH or GST alone, and the bound peptide was detected using anti-GST monoclonal antibody. We detected GST- δ-AmastinH (Fig. 3A) adhered to fixed or live HeLa cells in a saturable and dose-dependent manner (Fig. 3B). This finding suggests that amastin interacts with this host cell, probably through a putative receptor localized at the surface of HeLa cells, which suggested a role for amastin in the uptake of *T. cruzi* by HeLa cells.

### Amastin Inhibited Mammalian Cell Invasion

**Figure 4:**
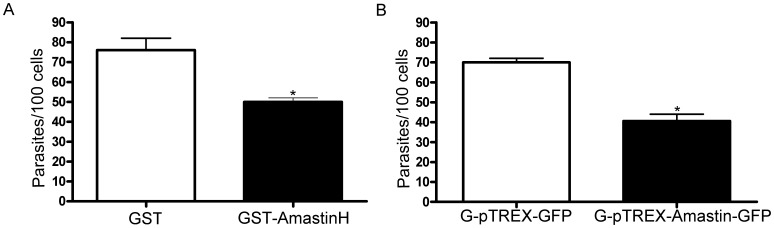
Delta-amastin interferes with EA-HeLa cell interaction. A: GST-AmastinH inhibited host cell invasion by *T. cruzi* EAs. Prior to invasion, HeLa cells were treated for 1 h with 5 µg/ml of GST (white column) or GST-δ-AmastinH (dark column). The parasites were then added to HeLa cells and the invasion proceeded for 2 h. The number of internalized parasites was counted in a total of at least 300 cells. **B: EAs (G strain) overexpressing amastin showed lower infectivity toward HeLa cells.** EAs of the G strain expressing high levels of δ-amastin (black column) showed a significant decrease in cell invasion when compared to wild type parasites (clear column) or parasites expressing only GFP (gray column). The invasion proceeded for 2 h. The values are shown as means ± standard deviations of two independent experiments performed in duplicate. *p<0.05.

After the amastin-derived hydrophilic polypeptide was shown to bind to host cells, we decided to investigate whether amastin could be important for parasite invasion. HeLa cells were pre-incubated in the presence of purified recombinant protein GST-δ-AmastinH or GST alone and then infected with EAs from the G strain. Whereas treatment with GST did not affect invasion, the GST-δ-AmastinH resulted in a 34% inhibition of parasite invasion (Fig. 4A).

G strain EAs transfected with Amastin-GFP (see description in the next section) also demonstrated a significant decrease in host cell invasion when compared to control EAs overexpressing GFP alone or the wild type parasites (Fig. 4B).

### Amastin Overexpression Triggered a Faster Metacyclogenesis

Since G strain EAs present low levels of amastin RNA compared to CL parasites (Fig. 1) we decided to increase expression of amastin by stable transfection of epimastigotes with pTREX-δ-Amastin-GFP plasmid. This plasmid carries a copy of amastin fused with Green Fluorescent Protein (GFP) under the control of the ribosomal protein TcP2β 5′UTR and glycosomal glyceraldehyde 3-phosphate dehydrogenase (gGAPDH) 3′UTR. The populations transfected with pTREX-GFP (control vector) or pTREX-δ-Amastin-GFP vectors were selected over two months with G418, and both populations reached high transfection efficiency as shown by flow cytometry analyses (>98% of G418 resistant parasites expressed GFP or Amastin-GFP, Fig. S3, supplementary material). The increase in amastin expression was quantified at the RNA level by qRT-PCR, and the correct translation was confirmed by western blot using whole cell extracts from transfected epimastigotes (Fig S4 and S5, supplementary material). It is noteworthy that real-time PCR revealed that the additional gene copy regulated by gGAPDH 3′UTR triggered ∼5 fold increase in δ-amastin mRNA levels in epimastigotes compared to pTREX-GFP parasites. The results shown in figure S4 also suggest that amastin expressed in fusion with GFP is not glycosylated since it migrates with the expected molecular size of an unmodified δ-Amastin/GFP fusion protein. Biochemical characterization of amastins indicate that they are present as large molecular weight glycoprotein complexes since they bind concanavalin A [20]. Although no conventional signals for N-linked glycosylation were identified, several serine and threonine residues that could be modified by 0-linked sugars are found in all amastin sequences. Parasite mobility, morphology (form, size and granularity) and growth rate were not affected by transfection (data not shown).

**Figure 5:**
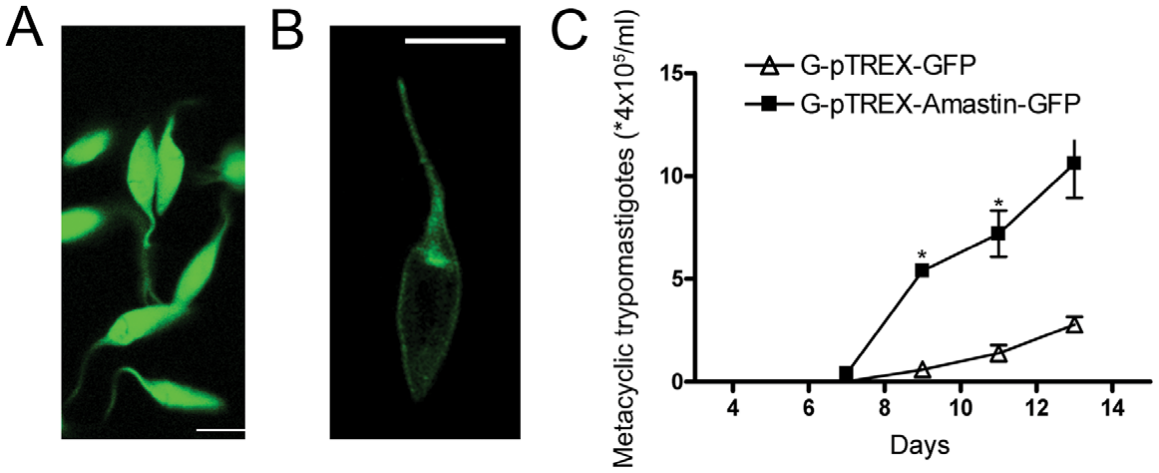
Transfection of G strain parasites with δ-amastin-GFP accelerates metacyclogenesis. A: G strain parasites were efficiently transfected with GFP and δ-amastin-GFP. A: Epimastigotes transfected with pTREX-GFP (G-pTREX-GFP) show a cytoplasmic diffuse fluorescence and **B:** epimastigotes transfected with pTREX-Amastin-GFP (G-pTREX-Amastin-GFP) display a surface fluorescence localization as well as a concentration near the kinetoplast and flagellum (B). (Bars = 3 µm). **C:** E**pimastigotes that overexpress** δ-**amastin displayed higher rates of metacyclogenesis.** A Neubauer chamber was used to evaluate the growth and differentiation rate of epimastigotes transfected with pTREX-GFP or pTREX-Amastin-GFP vectors. Standard deviations are derived from three replicates. *p<0.05.

Two months after transfection, we observed ∼95% of pTREX transfected epimastigotes expressing GFP (Fig. 5A) or δ-Amastin-GFP (Fig. 5B). To check if the amastin-GFP protein was being addressed to its correct subcellular destination, transfected epimastigote and amastigote forms were analyzed by confocal microscopy. The images shown in Figure 5 indicated that amastin fused with GFP localized at the surface and also concentrated in the vicinity of the kinetoplast, probably at the flagellar pocket region (Fig. 5B). This was a distinct and diffuse localization pattern from parasites transfected with GFP alone (Fig. 5A). Given that the fusion protein was being expressed at the expected location and in higher levels, these parasites were tested for phenotypic changes.

We observed that pTREX-δ-Amastin-GFP epimastigotes differentiated into metacyclic trypomastigotes faster than the control pTREX-G-GFP epimastigotes (Fig. 5C).

### Amastin Overexpression Also Accelerated the Differentiation of Amastigotes into TCTs

**Figure 6:**
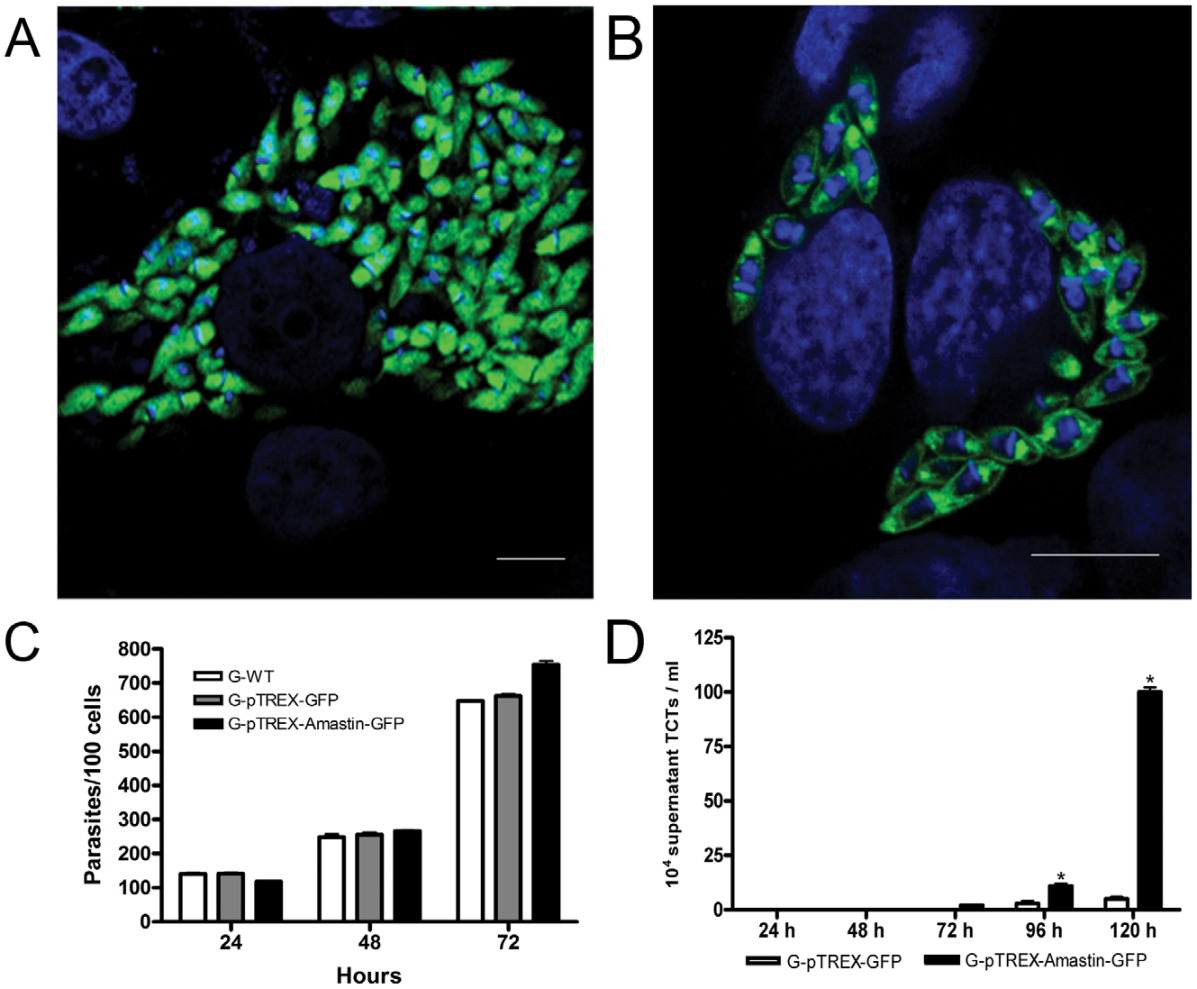
δ-amastin-transfected G-strain EAs transform more efficiently into trypomastigotes. A: EAs were efficiently transfected with pTREX-GFP and pTREX-Amastin-GFP. Vero cells were chronically infected with G-pTREX-GFP parasites (A) or with G-pTREX-Amastin-GFP parasites (**B**). A cytoplasmic fluorescence in A and a membrane localization of overexpressed amastins in **B** (bars = 10 µm)] were observed. DAPI (blue) showed nuclei and kinetoplasts staining. **C. The overexpression or δ-amastin does not affect intracellular growth of parasites.** Total number of intracellular amastigotes per 100 cell in infected HeLa cells were determined and it can be observed that overexpression of amastin does not interfere with the process. **D: Intracellular amastigotes overexpressing amastin transform faster and more efficiently into TCTs.** The number of TCTs at 96 h and 120 h after invasion was higher in the culture supernatants of cells infected with parasites expressing high levels of amastin (dark columns) in comparison to control cells infected with parasites transfected with pTREX-GFP (white columns). The values are shown as the means ± standard deviations of two independent experiments performed in duplicate. *p<0.05.

Full-length δ-amastin-GFP overexpressed in EAs of the G strain localized to the cell surface and also displayed a distinct concentration at the flagellar pocket (Fig. 6B), similar to the transfected epimastigotes (Fig 5B). A diffuse distribution pattern was observed when pTREX-GFP alone was expressed (Fig. 6A). Amastin overexpression did not affect intracellular growth of amastigotes (Fig. 6C). However, the overexpression of amastin in G strain EAs increased the numbers of trypomastigotes released into the supernatant of infected HeLa cells at 96 h and 120 h after invasion, as compared to the GFP controls (Fig. 6D). Intracellular trypomastigotes were also precociously observed in cells infected for 72 h with parasites overexpressing δ-amastin, and these forms were not observed in the control samples (figure S6).

### δ-Amastin Accelerated and Increased Parasite Tropism to Liver *in vivo*

**Figure 7:**
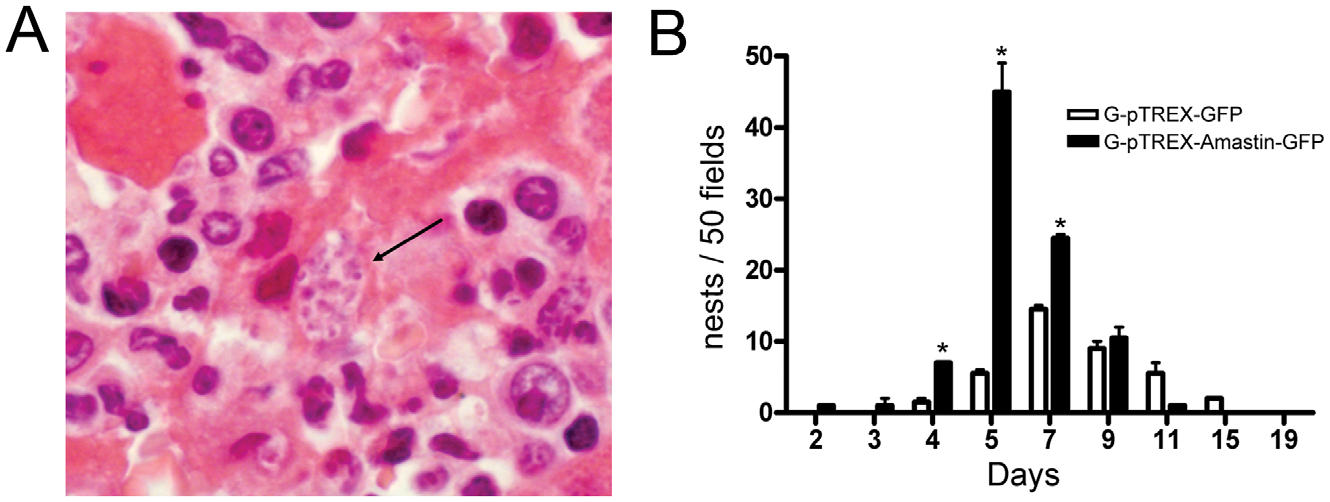
Transfection of G strain parasites with δ-amastin-GFP leads to early appearance of amastigote nests in the liver of infected mice. A. Nest of EAs (G-pTREX-Amastin-GFP) in the liver of infected A/JUnib mice. Susceptible mice (A/JUnib strain) were infected intravenously with 10^6^ G strain EAs transfected with pTREX-GFP or pTREX-Amastin-GFP. Nests of parasites overexpressing amastin were observed from the third day of infection. **B. Nests of G-pTREX-Amastin-GFP are formed earlier and in higher numbers in the liver of infected mice.** Thin sections of hematoxylin-eosin stained tissues from livers of mice infected with 5×10^6^ EAs from G-pTREX-GFP or G-pTREX-δ-Amastin-GFP strains were analyzed from the second day until the nineteenth day of infection for the determination of the number of parasite nests in the liver. The values are shown as means ± standard deviations of two independent experiments. *p<0.05.

We extended this investigation to an *in vivo* model of *T. cruzi* infection, inoculating A/JUnib mice with EAs transfected with GFP vector or overexpressing amastin-GFP. It has recently been shown that G strain extracellular amastigotes do not give rise to patent parasitemia in mice, possibly due to their susceptibility to interferon-gamma [35) In this study, bloodstream parasitemia was not detected in animals infected with G strain EAs either overexpressing pTREX-δ-Amastin-GFP or pTREX-GFP. Therefore, tissue parasitism following inoculation was assessed by counting amastigote nests in the liver (Fig. 7A). Nests of parasites overexpressing amastin were very early observed in the liver at the third day after inoculation and (Fig. 7B, dark bars). Amastigotes expressing empty GFP vector (G-pTREX-GFP, control) presented lower tissue parasitism from the 5th day that peaked at day 7 (Fig. 7B, white bars).

## Discussion

### Amastin as a Negative Regulator of *Trypanosoma cruzi* Cell Invasion

*Trypanosoma cruzi*, the etiological agent of Chagas’ disease, uses many strategies to invade mammalian cells. For instance, different infective stages (e.g. bloodstream trypomastigotes, metacyclic trypomastigotes and extracellular amastigotes), distinct strains and isolates, as well as differing infectivities, have been widely acknowledged as challenging tasks to overcome by researchers. Many researchers have set out to characterize the parasite proteins involved in the establishment of infection [13].

Very little is known about the molecules that participate in invasion of cells by EAs [13]. Possible candidates involved in cell invasion by EAs include members of the trans-sialidase superfamily and carbohydrate epitopes tagged to surface glycoproteins, as well as secreted components and cell-surface components [11], [12], [14], [18].

Here, we characterized one of the members of the previously identified amastin protein family of *T. cruzi* [20], a delta-amastin protein, examining its role during cell invasion and differentiation of parasites from the G strain.

Developmentally-regulated amastin proteins belong to one of the larger families of surface proteins in *Leishmania* [21] and show high similarity to the amastin proteins in *T. cruzi*. There are approximately 45 members of the amastin gene family and they are dispersed throughout the genome of *Leishmania* species. The structural organization of the amastin gene family in both *Leishmania* and *Trypanosoma* species indicate that they share a similar structural organization and contain a highly conserved 11 amino acid extracellular domain, unique to amastin proteins [21]. Hydrophobicity profiling predicted four transmembrane helices for the majority of the amastin homologs and this strongly suggested membrane localization for these proteins [21]. This was confirmed at the subcellular level for three amastin gene products of *Leishmania* [21]. Studies on the evolution and diversification of this family suggest that amastin, which suffered a major diversification after the origin of the genus *Leishmania*, can be subdivided into four groups: alpha, beta, delta and gamma-amastin. Alpha- and gamma- amastin have been only identified in *Leishmania* spp and in the insect trypanosomatid *Crithidia deanei.* There are two copies of beta-amastin organized in tandem present in the genomes of *Leishmania* spp, *Crithidia* spp and *T. cruzi* [24].

Delta-amastin belongs to the sub-family with the highest number and diversity among its members. There are multiple copies of delta-amastin genes present in the genomes of *Leishmania* spp, *Crithidia* spp and *T. cruzi* arranged in clusters in which amastin genes are interspersed with tuzin genes. The delta sub-family underwent an expansion in the genus *Leishmania*, and in *T. cruzi* could still be sub-divided into two groups named delta and proto-delta amastin. In the *T. cruzi* genome two copies of proto-delta amastin genes are present in the chromosome 26 whereas at least 20 copies of delta-amastin genes are present in the chromosome 34 [24]. We have chosen one member of the delta-amastin sub-family in this study to be over-expressed in the G strain because the mRNA expression analysis showed that, contrary to *T. cruzi* beta-amastin genes, the expression of delta-amastin genes is largely reduced in the G strain [20]. Because all delta-amastin genes have over 92% of amino acid identity, the member we selected for over-expressing in the G strain would be representative of all members from this group.

We have been able to express and purify a hydrophilic and immunogenic region of *T. cruzi* delta-amastin and raise specific antibodies to this molecule. As previously demonstrated for *Leishmania* [21] and *T. cruzi* [20], amastin is localized at the amastigote surface. The transcripts of amastin have been detected by microarray (not shown), qRT-PCR and northern blot assays. By comparing the total quantity of transcripts between G and CL strains, the amount of amastin transcripts was always higher in the CL than the G strain. Regarding EAs as infective forms, it has been known that the *T. cruzi* G strain is more infective to HeLa and other mammalian cells than the CL strain [35]–[40]. Given that amastigotes from the CL strain contained more transcripts and amastin mRNA than the G strain and CL EA parasites are less infective [11], we hypothesized that amastin could be a negative regulator of amastigote cell invasion, in line with previous observations of metacyclic trypomastigotes [6].

Our results showed that the amastin displayed properties required for a protein associated with host cell invasion. First, the protein is expressed on the amastigote surface, and the truncated recombinant form adhered to host cells in a dose-dependent manner, both properties observed in previously characterized *T. cruzi* proteins [6], [14]. Next, the treatment of host cells with recombinant amastin led to a significant decrease in parasite internalization. Possibly, the contact of the hydrophilic region with a putative receptor interferes with a key process that inhibits invasion of HeLa cells. These results also showed that the binding region involved in cell adhesion and invasion is located within the amastin domain cloned and expressed in this study. A novel set of truncated recombinant proteins would help to clarify and narrow down the required amino acid sequence of amastin that regulates cell adhesion and invasion. Thus, taken together these results collectively point to a role of amastin in modulating cell invasion by *T. cruzi*.

### The Role of Amastin in Parasite Intracellular Survival

Some indirect evidence has pointed to a crucial role of amastin in the co-evolution, adaptation and survival of trypanosomatids within both vertebrate and invertebrate hosts. For instance, amastin proteins seem to operate at the host-parasite interface and are implicated in severe disease, as they evoke strong immune responses in both mice and humans, primarily when associated with visceral leishmaniasis [25], [26]. Comparative analysis indicated that the amastin family is both diverse and ancient [24]. The emergence of the amastin family pre-dates the diversification of the various parasite species, allocating amastin as an ancient feature of all trypanosomatid genomes [24]. This suggests a preparation of the trypanosomatids in the course of evolution to develop such specialized molecules to support both survival and prevalence of trypanosomatids in a hostile environment. In addition, the putative role of amastin in the intracellular survival of the parasite has been suggested by DNA microarray analysis data [27]. Amastin genes were predominantly expressed in amastigotes of different *L. donovani* strains isolated from post-kala-azar dermal leishmaniasis and visceral leishmaniasis patients, and this was also shown for laboratory strains [28].

Other indirect observations corroborate a role for amastin in intracellular survival. Some authors have questioned whether amastin genes contribute to parasite pH homeostasis and growth inside the parasitophorous vacuole. As transmembrane proteins, amastins could play a role in proton or ion traffic across the membrane [21], [41]. Interestingly, a number of amastin homologs are expressed once the parasite is fully differentiated into its intracellular amastigote form, a process that normally occurs inside the acidic environment of phagolysosomes [21]. Opsonization of *Leishmania* amastigotes via host IgG antibodies promotes its uptake via the macrophage Fc receptors and the release of IL-10, which favors amastigote proliferation by changing the metabolism of the host macrophage. Amastin is considered to be the candidate for this surface epitope recognized by these opsonic antibodies [42].

Here, we have conclusively demonstrated for the first time that amastin may have a role in differentiation of *T. cruzi* both *in vitro* and *in vivo*. First, overexpression of amastin in *T. cruzi* increases epimastigote differentiation into metacyclic trypomastigotes, all of which are stages residing in the insect vector. Second, amastigotes that overexpress δ-amastin, although not growing at higher rates if compared to the controls, are able to differentiate much faster into trypomastigotes within infected cells and tissues, which are the stages exclusive to the mammalian host. Finally, the infection of mice with EAs overexpressing amastin triggers an increased and premature tropism to the liver. Taken together our results show that amastin is a key molecule responsible for the survival of *T. cruzi* in its intracellular cell stage.

### Amastin and the Trade-off Hypothesis

It is apparent from the data presented here as well as through indirect evidence presented by other authors, that amastin facilitates parasite survival by accelerating the generation of infective trypomastigotes, ensuring parasite persistence and spreading within the host. Amastin is likely to be a molecule that aids the parasite to maximize transmissibility which suggests a behavior that coincides with the so- called “trade-off hypothesis.” The trade-off hypothesis, developed by Anderson & May [43] and Ewald [44], takes into account that there are both fitness benefits and costs associated with virulence. If a parasite kills its host, it also kills itself and as a consequence prevents its further transmission. In this case, the host death is assumed to be “the cost,” because in theory parasites evolve to be relatively benign with no benefit from killing their host. Conversely, the generation of greater numbers of transmissible forms per unit time, and/or the increased persistence in a live host could be the benefits associated with virulence [45], [46].

One could argue that there are both positive and negative selective forces in nature acting on virulence and that virulence is a property of a host-parasite interaction, and not simply of the parasite. Long-term parasite-host associations are the result of an advantageous coevolution since their interaction has been long enough for adaptation. To balance the fitness cost to the pathogen when the host dies as a result of infection, it is assumed that there must also be a virulence-related advantage to the pathogen’s fitness [45], [46].

**Figure 8:**
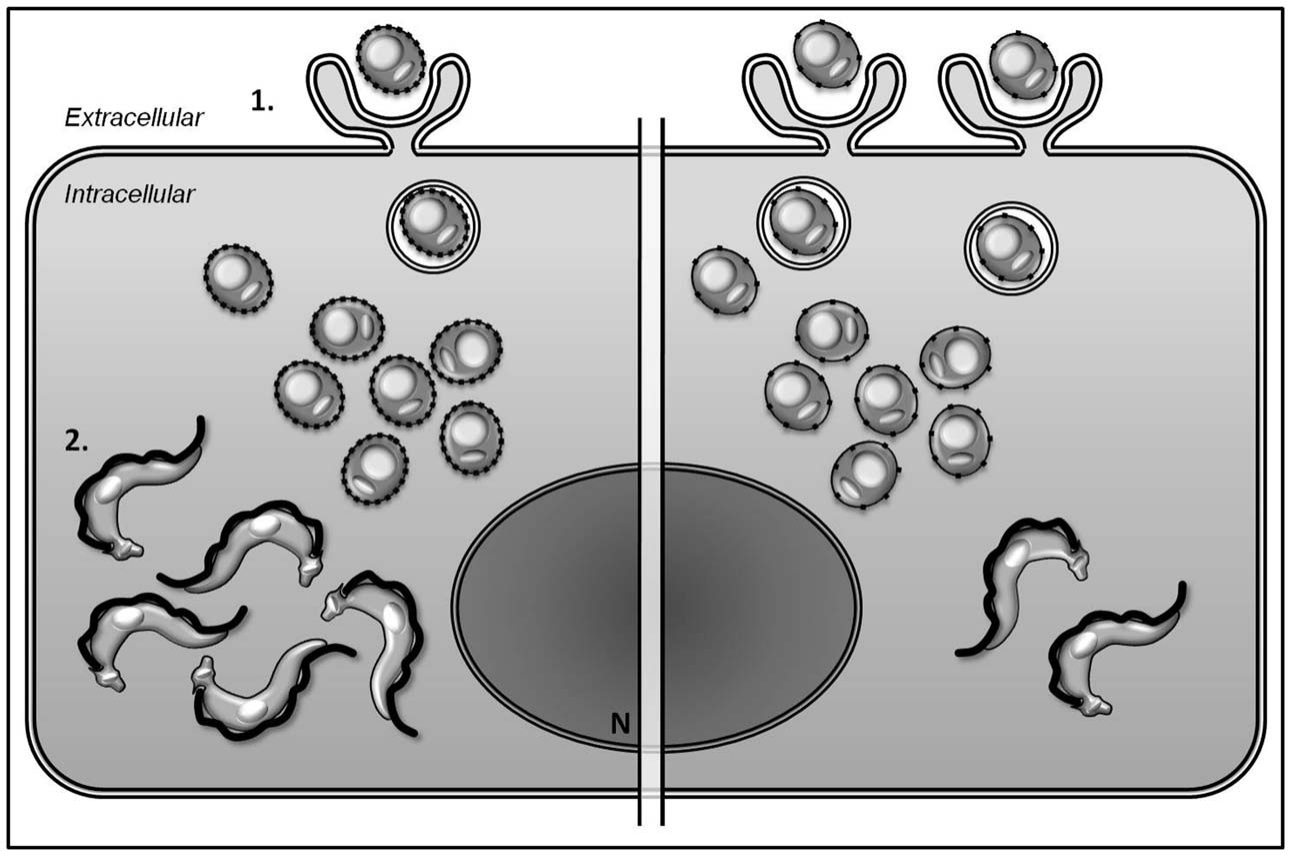
Schematic proposed model for the role of δ-amastin in *T. cruzi* virulence. The model indicates two distinct parasites, EAs expressing high levels of amastin was represented by dotted line membrane of amastigotes (left cell side) whereas the low amastin expression was represented by spaced dots in the amastigotes membrane (right cell side), during EA invasion and differentiation processes of *T. cruzi* virulence in a hypothetical host cell. **1.** EAs expressing more amastin (left) show a lower infectivity rate when compared with parasites expressing lower levels of the protein. **2.** High levels of amastin accelerate the transformation of amastigotes into TCTs (left).

Going further in the case of *T. cruzi* and amastin, it seems that the cost for the parasite would be to be less infective, i.e. fewer parasites (EAs) enter into host cells, and this could be an alternative strategy to avoid recognition by the immune system which, in turn, would ultimately damage the host. By contrast amastin allows faster differentiation into a new round of infective forms (see proposed model, Figure 8). Pathogens with the highest fitness are those with an intermediate level of virulence, which balances these opposing contributions to fitness. Taking the behavior of amastin described above into account, this may be the case in relation to *T. cruzi* virulence.

## Supporting Information

Figure S1 **Specificity of anti-recombinant GST-δ-AmastinH antibodies by immunofluorescence.** A–C mixtures of *T. cruzi* epimastigotes (red arrow) and trypomastigotes (yellow arrow) of the CL strain do not react with anti-recombinant GST-δ-AmastinH. A; DIC image. B: DAPI image; C: anti-recombinant GST- δ-AmastinH image. D–F: CL strain extracellular amastigotes react with the anti-recombinant GST-δ-AmastinH: by comparison, in the same experiments, EA (also CL strain) are fully labeled: D: DIC; E: DAPI image; F: anti-recombinant GST-δ-AmastinH image. Bar = 10 µm.
(TIF)

Figure S2 **Specificity controls on western blots. A: anti-GST:** 1) 4 µg of recombinant GST- δ-AmastinH; 2) 20 µg of GST. **B: anti-GFP:** 1) Total extract of epimastigote of G_pTREX-Amastin-GFP; 2: Total extract of epimastigote of G_pTREX-GFP; 3) Total extract of WT G strain epimastigote. **C: anti- GST-** **δ-AmastinH:** 1) 1) Total extract of epimastigote of G_pTREX-Amastin-GFP; 2: Total extract of epimastigote of G_pTREX-GFP. **D: Left Panel: anti- GST-δ-AmastinH:** 1) GST, 20 µg; 2) 4 µg of recombinant GST-δ-AmastinH; 3) Total extract of WT EA of CL strain; 4) Total extract of WT epimastigotes of CL strain; **5)** Total extract of WT EA of G strain; 6) Total extract of WT epimastigote of G strain. **Right Panel:** Coomassie loading control of D.
(TIF)

Figure S3 **Flow cytometry analysis showing high level of transfection efficiency.** G, G-pTREX-GFP and G-pTREX-δ-Amastin-GFP epimastigotes were washed with cold PBS and analyzed using a BD FACSCalibur® flow cytometer (Becton Dickinson) with 10^4^ gated events acquired for analysis. G-pTREX-GFP (green curve) and G-pTREX-Amastin-GFP (red curve) showed homogeneous populations with transfection rates >98%. Untransfected control parasites (G strain), black curve.
(TIF)

Figure S4 **The** **relative amount of amastin mRNAs in epimastigotes transfected with pTREX-Amastin-GFP was higher than in epimastigotes transfected with pTREX-GFP.** Transcript levels were determined by quantitative real-time PCR using SYBR® Green I chemistry. Quantitative real-time PCR was performed on RNA samples from epimastigotes of G-pTREX-GFP and G-pTREX-δ-Amastin-GFP strains. The comparative mRNA levels were determined after normalization with GAPDH amplicons. Standard deviations are derived from three replicates (*p<0.05).
(TIF)

Figure S5 **Immunoblot analysis of transfected epimastigotes.** G, G-pTREX-GFP or G-pTREX-δ-Amastin-GFP cell lysates were prepared by homogenization of cell pellets in Laemmli sample buffer, separated by 12.5% standard SDS-PAGE, transferred to Hybond-C membranes and incubated with mouse anti-GFP followed by secondary antibody.
(TIF)

Figure S6 **Intracellular trypomastigotes are detected after 72 h in cells infected with parasites superexpressing δ-amastin.** HeLa cells infected with A: WT, B: GFP or C: GFP-amastin; cells were fixed with Bouin and stained with Giemsa for the determination of intracellular parasite growth.
(TIF)
