# Correction: *Trypanosoma cruzi*: Role of δ-Amastin on Extracellular Amastigote Cell Invasion and Differentiation

**DOI:** 10.1371/annotation/62f40c30-85c4-4de0-a019-6e0b30cc394a

**Published:** 2013-10-04

**Authors:** Mário C. Cruz, Normanda Souza-Melo, Claudio Vieira da Silva, Wanderson Duarte DaRocha, Diana Bahia, Patrícia R. Araújo, Santuza R. Teixeira, Renato A. Mortara

There were errors in the legend of Supporting Information Figure S2. The correct text of that legend is available below.

Figure S2

Specificity controls on western blots. A: anti-GST: 1) 10 µg of recombinant GST- δ-AmastinH; 2) 10 µg of GST. B: anti-GFP: 1) Total extract of epimastigote of G_pTREX-Amastin-GFP; 2: Total extract of epimastigote of G_pTREX-GFP; 3) Total extract of WT G strain epimastigote. C: anti- GST- δ-AmastinH: 1) 10 µg of recombinant GST- δ-AmastinH; 2) 10 µg of GST. D: Left Panel: anti- GST-δ-AmastinH: 1) 10 µg, GST; 2) 4 µg of recombinant GST-δ-AmastinH; 3) Total extract of WT EA of CL strain; 4) Total extract of WT epimastigotes of CL strain; 5) Total extract of WT EA of G strain; 6) Total extract of WT epimastigote of G strain.

Right Panel: Coomassie loading control of D. 

