# Supplementary figures and images for: Trypanosoma cruzi: Role of δ-Amastin on Extracellular Amastigote Cell Invasion and Differentiation
Source: PLoS One. 2012 Dec 18;7(12):e51804. doi: 10.1371/journal.pone.0051804 (PMC3525664; doi:10.1371/journal.pone.0051804)

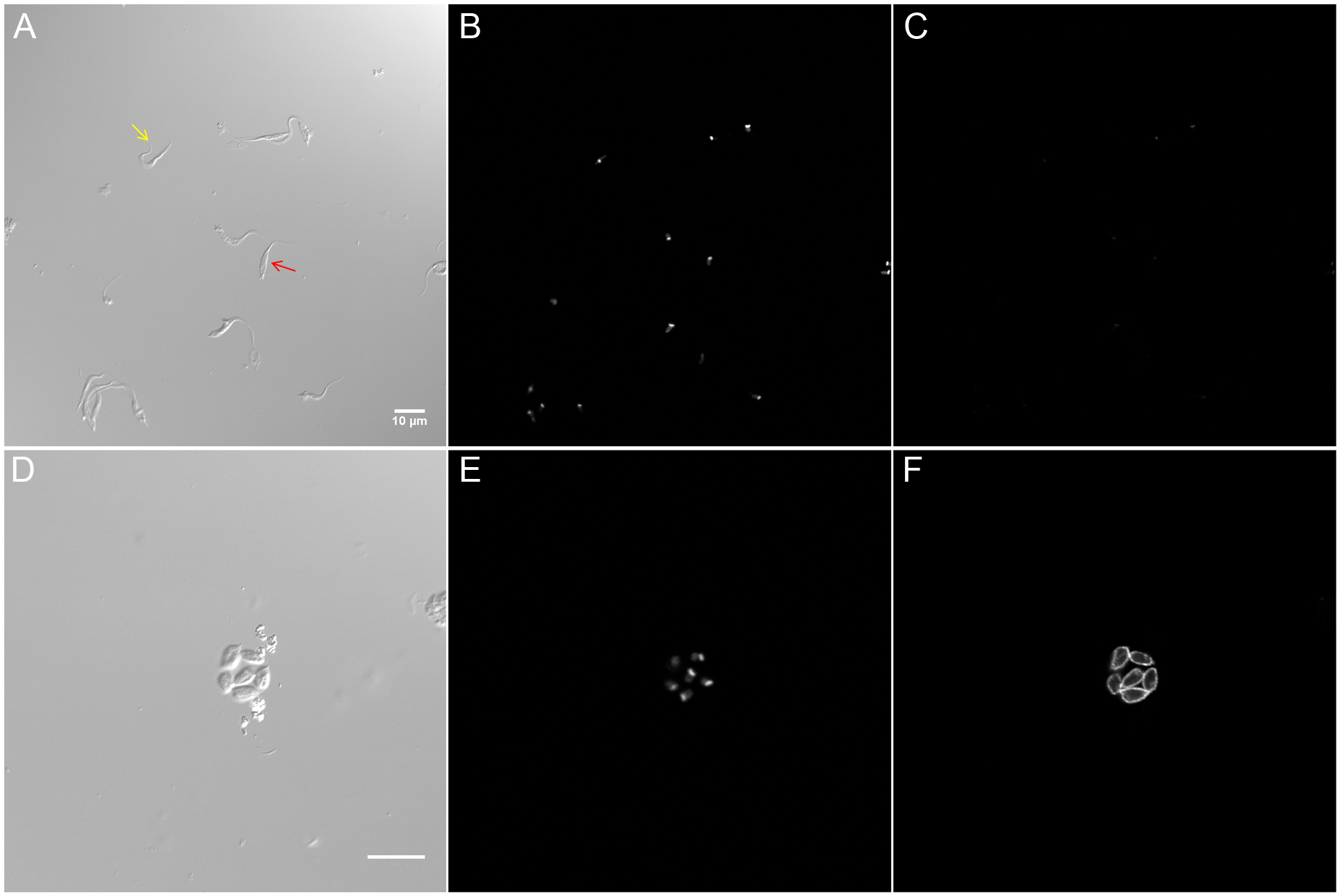

Supplement: Figure S1 — Specificity of anti-recombinant GST-δ-AmastinH antibodies by immunofluorescence. A–C mixtures of T. cruzi epimastigotes (red arrow) and trypomastigotes (yellow arrow) of the CL strain do not react with anti-recombinant GST-δ-AmastinH. A; DIC image. B: DAPI image; C: anti-recombinant GST- δ-AmastinH image. D–F: CL strain extracellular amastigotes react with the anti-recombinant GST-δ-AmastinH: by comparison, in the same experiments, EA (also CL strain) are fully labeled: D: DIC; E: DAPI image; F: anti-recombinant GST-δ-AmastinH image. Bar = 10 µm. (TIF) [file pone.0051804.s001.tif]

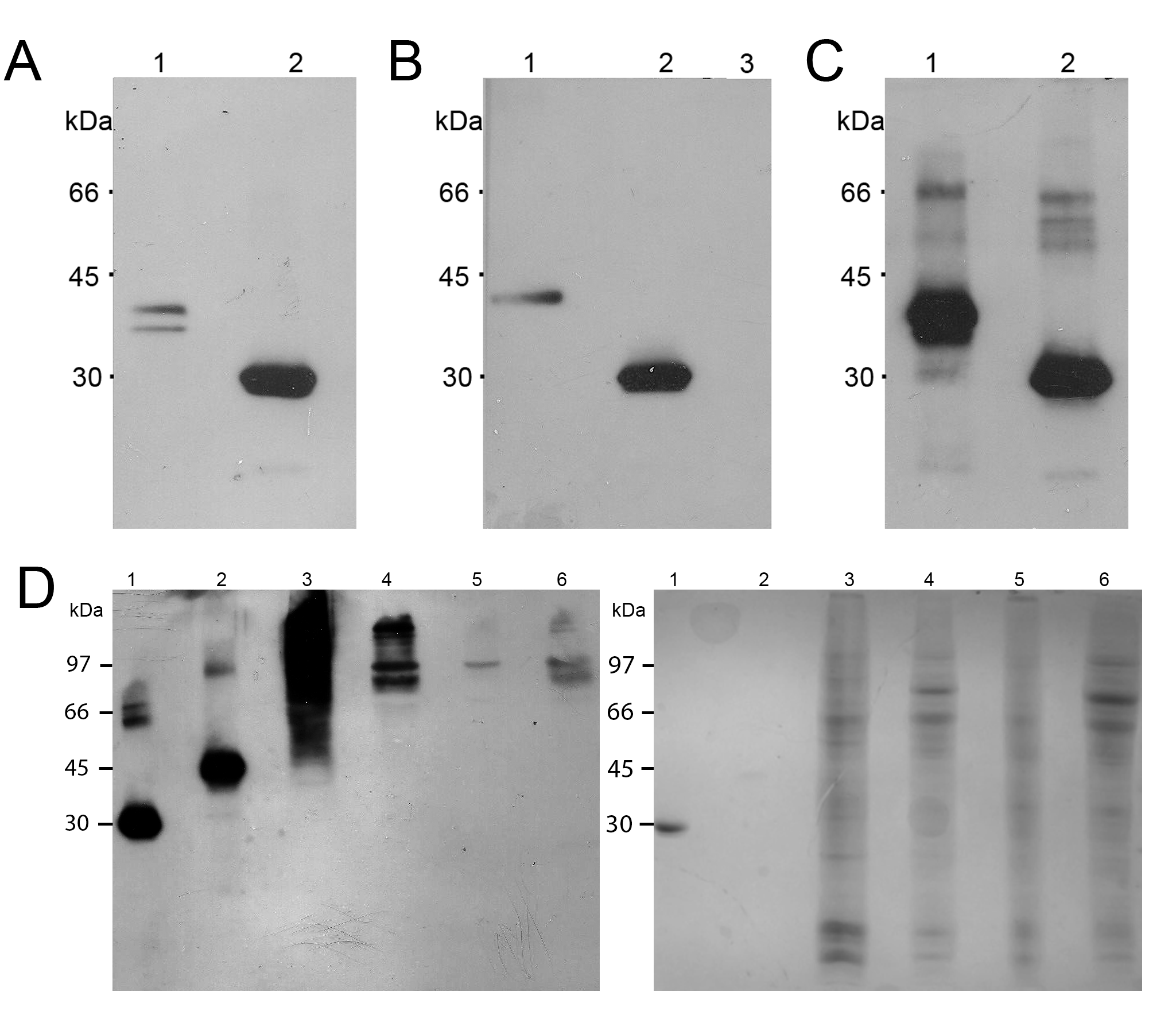

Supplement: Figure S2 — Specificity controls on western blots. A: anti-GST: 1) 4 µg of recombinant GST- δ-AmastinH; 2) 20 µg of GST. B: anti-GFP: 1) Total extract of epimastigote of G_pTREX-Amastin-GFP; 2: Total extract of epimastigote of G_pTREX-GFP; 3) Total extract of WT G strain epimastigote. C: anti- GST- δ-AmastinH: 1) 1) Total extract of epimastigote of G_pTREX-Amastin-GFP; 2: Total extract of epimastigote of G_pTREX-GFP. D: Left Panel: anti- GST-δ-AmastinH: 1) GST, 20 µg; 2) 4 µg of recombinant GST-δ-AmastinH; 3) Total extract of WT EA of CL strain; 4) Total extract of WT epimastigotes of CL strain; 5) Total extract of WT EA of G strain; 6) Total extract of WT epimastigote of G strain. Right Panel: Coomassie loading control of D. (TIF) [file pone.0051804.s002.tif]

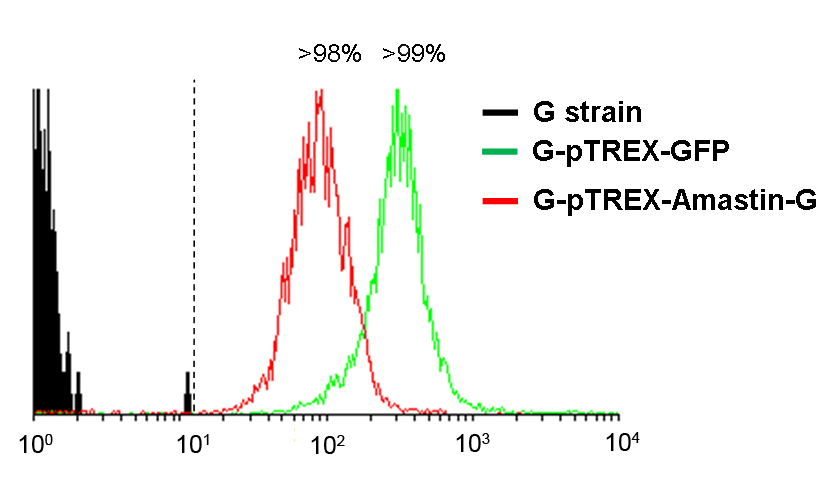

Supplement: Figure S3 — Flow cytometry analysis showing high level of transfection efficiency. G, G-pTREX-GFP and G-pTREX-δ-Amastin-GFP epimastigotes were washed with cold PBS and analyzed using a BD FACSCalibur® flow cytometer (Becton Dickinson) with 104 gated events acquired for analysis. G-pTREX-GFP (green curve) and G-pTREX-Amastin-GFP (red curve) showed homogeneous populations with transfection rates >98%. Untransfected control parasites (G strain), black curve. (TIF) [file pone.0051804.s003.tif]

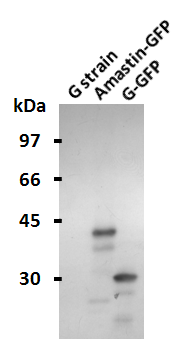

Supplement: Figure S5 — Immunoblot analysis of transfected epimastigotes. G, G-pTREX-GFP or G-pTREX-δ-Amastin-GFP cell lysates were prepared by homogenization of cell pellets in Laemmli sample buffer, separated by 12.5% standard SDS-PAGE, transferred to Hybond-C membranes and incubated with mouse anti-GFP followed by secondary antibody. (TIF) [file pone.0051804.s005.tif]

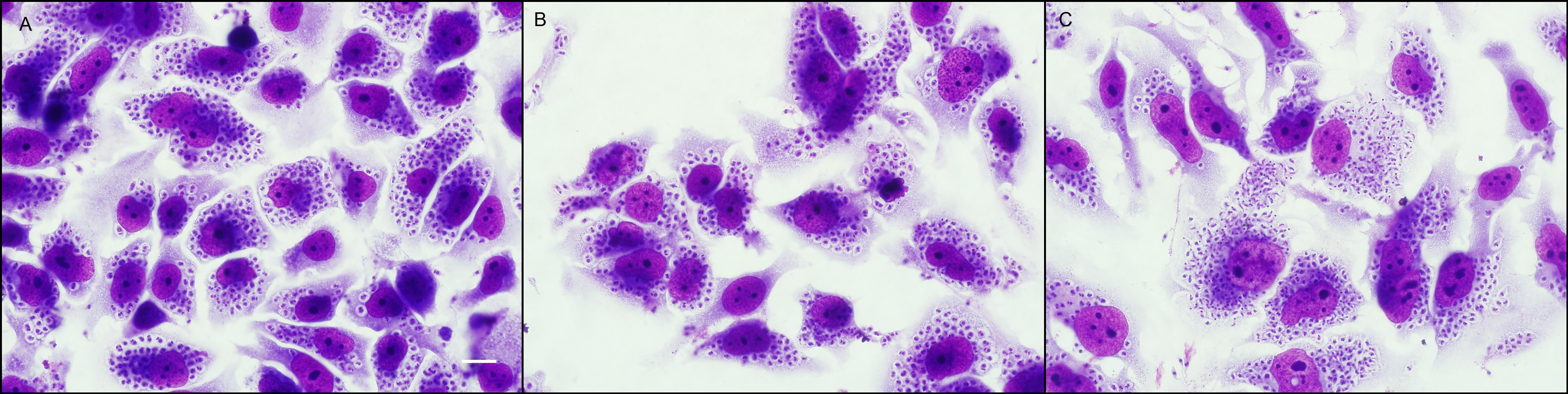

Supplement: Figure S6 — Intracellular trypomastigotes are detected after 72 h in cells infected with parasites superexpressing δ-amastin. HeLa cells infected with A: WT, B: GFP or C: GFP-amastin; cells were fixed with Bouin and stained with Giemsa for the determination of intracellular parasite growth. (TIF) [file pone.0051804.s006.tif]
